# The Extent of Misconceptions, Negative Prejudices, and Discriminatory Behaviors Toward Psoriasis Patients: A Cross-Sectional Survey Study Among the Population of Jeddah, Saudi Arabia

**DOI:** 10.7759/cureus.41678

**Published:** 2023-07-11

**Authors:** Rakan Abu Alqam, Haya Obaid, Raghad Aljondi, Badr Alsulymani, Raghad Aljohani, Saud M Aleissa, Ahmed Baabdullah, Bader S Zimmo, Mohammed H Abduljabbar, Jehad Hariri

**Affiliations:** 1 Medicine, King Abdulaziz University Faculty of Medicine, Jeddah, SAU; 2 Medical School, King Abdulaziz University Faculty of Medicine, Jeddah, SAU; 3 Dermatology, King Abdulaziz University Hospital, Jeddah, SAU; 4 Dermatology, King Abdulaziz University Faculty of Medicine, Jeddah, SAU

**Keywords:** behaviors, negative prejudice, misconception, perception, psoriasis

## Abstract

Background

Psoriasis is a chronic inflammatory disease that affects around 2% of the population. The lives of psoriasis patients are greatly impacted by stigma and social exclusion, regardless of the severity of the condition. This is the first study of its kind to be conducted in Saudi Arabia. This study aimed to assess peoples’ psoriasis-related misconceptions, negative prejudice, and discriminatory behaviors.

Methodology

A self-administered Google Forms survey was distributed between January and February 2023. The survey was conducted among residents of Jeddah, Saudi Arabia who were enrolled randomly via social media. We aimed to investigate their perception and awareness related to psoriasis, as well as how varying educational levels, age groups, and genders affected these variables.

Results

In total, 803 individuals participated in the survey. Results showed that 19.9% of the participants did not know about psoriasis. Only 5.1% of respondents stated that they were well knowledgeable about psoriasis. Moreover, psoriasis was more frequently perceived as a communicable disease by people in the age group of 18-29 years (p = 0.000). Surprisingly, only 43.5% of the participants reported that they would shake hands with someone with psoriasis without hesitation. Additionally, 40.7% of the participants were aware that psoriasis requires lifelong treatment. Moreover, the belief that psoriasis does not require lifelong therapy was more prevalent among university graduates (p = 0.000).

Conclusions

This study found that the residents of Jeddah need further education on skin disorders, in general, and psoriasis, in particular. Future studies should be conducted utilizing various approaches, with a greater emphasis on certain groups of people who engage physically with psoriasis patients.

## Introduction

Psoriasis is a chronic inflammatory illness that affects around 2% of the population and is characterized by erythematous scaly plaques that can vary in severity from a few scattered red, scaly plaques to nearly complete body surface involvement [1]. Previous infections, certain drugs, and emotional and physical stress can have a negative impact on the etiology of the disease [2]. Furthermore, a lack of social relationships can lead to issues such as emotional tension [3]. Psoriasis patients receive less social support and face more stigma than those with other dermatological illnesses [4,5]. Patients are frequently stigmatized as psoriasis lesions are noticeable on the face, scalp, and hands [6,7]. Patients with psoriatic lesions in easily visible locations, such as the back of the hand, may experience stigma more frequently and may require more effective therapies [8]. A large-scale study found that, in addition to those with lesions on the hands, face, and scalp, patients with psoriatic arthritis and inverse psoriasis who do not have lesions on visible areas may frequently experience internalized stigma and this feeling may form the basis of the psychological and social burden of psoriasis [9]. Furthermore, it has been reported that psoriasis patients have low self-esteem and extreme anxiety, underlining the need for family and social support [10]. Psoriasis patients’ lives are negatively impacted by stigma and social isolation, which increases their risk of depression and anxiety and leads to a lower health-related quality of life regardless of disease severity [11,12]. Several studies have been conducted to explore the impact of stigmatizing behaviors on psoriasis patients [6,13]. However, few studies have examined the community’s psoriasis perception and actions. To our knowledge, this is the first study of its kind from Saudi Arabia. This study aimed to assess individuals’ psoriasis perception, attitudes, and behaviors, as well as the influence of varying levels of education, age, and gender on knowledge, attitudes, and actions.

## Materials and methods

Study design and data collection

This cross-sectional study was conducted via an online Google Forms questionnaire from January to February 2023. A self-administered survey was distributed randomly to people living in Jeddah, Saudi Arabia through social media platforms, including WhatsApp, Telegram, Instagram, Twitter, and others to evaluate individuals’ psoriasis perceptions and behaviors. In addition, the survey assessed the impact of different levels of education, age, and gender on awareness. The questionnaire reached 803 participants. All participants were notified about the preconditions of the study and assured of the confidentiality of their responses. Informed consent was obtained from all participants.

Questionnaire variables

The questionnaire was developed based on our study’s objectives and an accessible questionnaire with similar objectives [14]. The questionnaire had 12 questions divided into the following six sections: demographics, experience of psoriasis, knowledge, misconceptions, behavior, and negative prejudice. The first section inquired about age, gender, education level, and income level. The second section aimed to assess the experience with psoriasis, which involved the following questions: “have you heard of psoriasis,” “have you ever had psoriasis,” and “do you know anyone who has psoriasis.” The third section included questions about the level of knowledge. The fourth section was designed to assess the misconception through statements classified as “true,” “not sure,” and “false.” The fifth and sixth sections aimed to assess the behavior and negative prejudices, respectively, through a five-point Likert scale. Participants had the option to select “do not know.”

Statistical analysis

Statistical analysis was performed using SPSS version 21.0 (IBM Corp., Armonk, NY, USA). The demographics, descriptive characteristics of respondents, and correct answers to questions about psoriasis were presented using descriptive statistics. The association between demographics, misconceptions, behaviors, and negative prejudices was determined using a descriptive one-way analysis of variance test. To assess experience level, responses were categorized as “yes” (experienced), “no” (not experienced), and “I don’t know.” Responses to the self-assessed level of knowledge were divided into the following two categories: “well informed” (involving very well and relatively well informed) and “not well informed” (involving all other responses). Misconceptions were evaluated through sentences, and the responses were divided into “true,” “false,” and “not sure.” Responses to items for behavior assessment were categorized as “yes” (I would do it without any reservations) and “no” (all other responses). While responses to items evaluating negative prejudices were divided into “agree” (involving completely agree and mostly agree) and “disagree” (including completely disagree, do not know, and mostly disagree). Statistical significance was defined as p-values <0.05. Data analysis was performed with 95% confidence intervals.

Ethical consideration

Ethical approval was obtained from the Unit of Biomedical Ethics Research Committee at the Faculty of Medicine, King Abdulaziz University, Jeddah, Saudi Arabia (reference number: 441-22).

## Results

A total of 803 participants were included in our study through an online survey. Participants’ mean age was 34.19 ± 14.84 years. The vast majority of participants were females 565 (70.4%). In total, 576 (71.7%) were university graduates. More than half of the participants’ income levels per month were less than 10,000 SAR (Table 1).

**Table 1:** Demographic characteristics of the participants.

	Frequency	Percent
Age group (years)		
<18	48	6%
18–29	354	44.1%
30–39	94	11.7%
40–49	128	15.9%
50–60	154	19.2%
>60	25	3.1%
Mean	34.192 ± 14.8459	
Gender		
Male	238	29.6%
Female	565	70.4%
Education level		
University	576	71.7%
High school	205	25.5%
Intermediate school	20	2.5%
Elementary	2	0.2%
Monthly income		
Less than 10000 SAR	441	54.9%
10000–20000 SAR	278	34.6%
21000–30000 SAR	56	7%
More than 30000 SAR	28	3.5%

**Figure 1:**
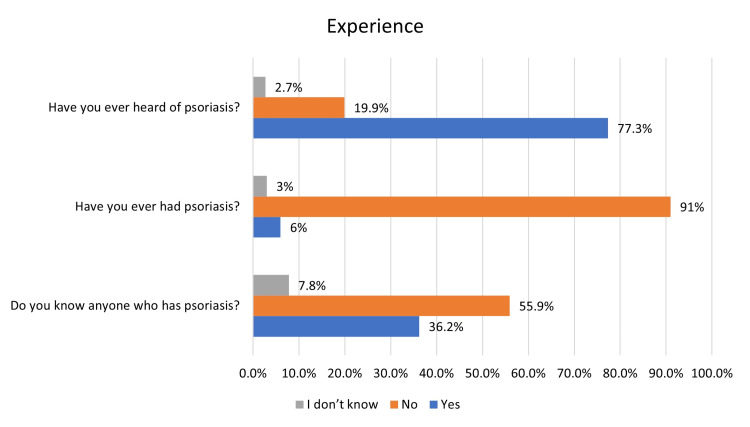
Participants’ experience with psoriasis.

**Figure 2:**
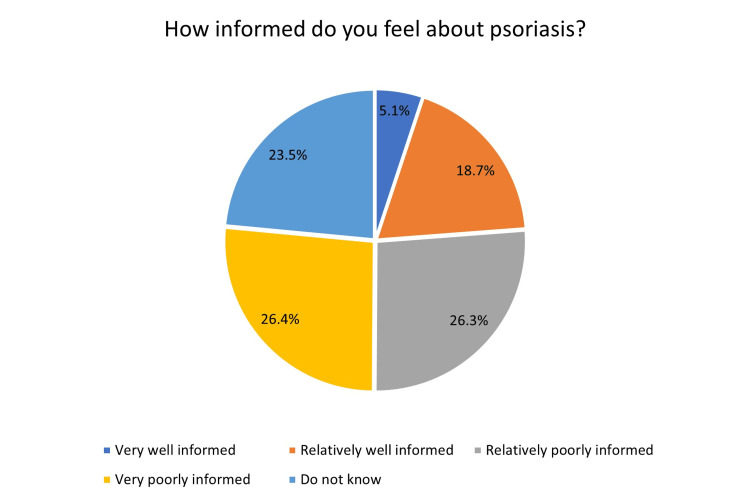
Participants’ knowledge of psoriasis.

About 19.9% of participants were not aware of psoriasis, yet only 6% of the participants had psoriasis. However, 36.2% knew someone with psoriasis (Figure 1). Regarding the participant’s information about psoriasis, surprisingly, only 5.1% believed that they were well-informed about psoriasis, while 18.7% thought that they were relatively well-informed. In addition, 26.3% assumed that they were relatively poorly informed, and 26.4% thought that they were poorly informed. Finally, 23.5% believed that they knew nothing to be mentioned (Figure 2).

**Figure 3:**
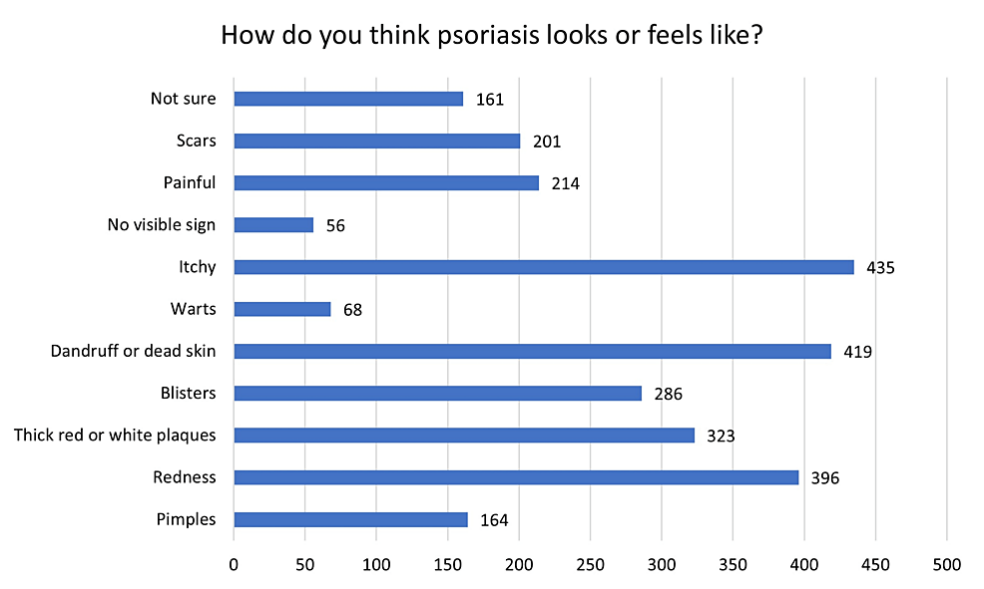
Responses to the question “How do you think psoriasis looks or feels like?”

In a multiple-answer question, 435 participants reported that psoriasis feels itchy, and 419 individuals reported that psoriasis looks like dandruff and dead skin. Moreover, 396 participants stated that psoriasis makes the skin red, and only 323 participants chose thick red or white plaques (Figure 3). Meanwhile, only 40.7% of the participants knew that psoriasis needs lifelong treatment. Further, only 32.5% knew that psoriasis is a genetic condition. Additionally, only 31.5% knew that psoriasis can affect the joints (Table 2). Surprisingly, only 43.5% would shake hands with someone with psoriasis without reservations. Moreover, 34.5% were willing to eat a meal prepared by someone with psoriasis, and 10.1% would forbid their children from playing with someone suffering from psoriasis (Table 3).

**Table 2:** Participants’ misconceptions about psoriasis.

	Correct answer	Frequency	Percent
Psoriasis is contagious			
True	False	76	9.5%
False		493	61.4%
Not sure		234	29.1%
Psoriasis is due to poor hygiene			
True	False	63	7.8%
False		537	66.9%
Not sure		207	25.3%
Psoriasis can be treated			
True	True	527	65.6%
False		111	13.8%
Not sure		165	20.5%
Psoriasis needs life-long treatment			
True	True	327	40.7%
False		162	20.2%
Not sure		314	39.1%
Psoriasis is a serious condition			
True	False	141	17.6%
False		423	52.7%
Not sure		239	29.8%
Psoriasis is a genetic condition			
True	True	261	32.5%
False		141	17.6%
Not sure		401	49.9%
Psoriasis can affect joints			
True	True	253	31.5%
False		157	19.6%
Not sure		393	48.9%

**Table 3:** Participants’ behaviors toward psoriasis.

	I would do it without any reservations	I would do it with reservations	I would really hesitate to do it	I would refuse to do it	Do not know
Shake hands with a person who has psoriasis	349 (43.5%)	220 (27.4%)	102 (12.7%)	48 (6%)	84 (10.5%)
Eat a meal prepared by someone with psoriasis	277 (34.5%)	189 (23.5%)	146 (18.2%)	107 (13.3%)	84 (10.5%)
Be friends with someone with psoriasis	465 (57.9%)	161 (20%)	73 (9.1%)	33 (4.1%)	71 (8.8%)
Sit next to someone with psoriasis	450 (56%)	169 (21%)	76 (9.5%)	37 (4.6%)	71 (8.8%)
Let my children play with someone with psoriasis	344 (42.8%)	177 (22%)	109 (13.6%)	81 (10.1%)	92 (11.5%)

On the other hand, regarding negative prejudice, 25.9% of participants agreed to some extent that people with psoriasis tend to take more sick leaves. Moreover, 22% completely disagreed that having psoriasis would be a worry while applying for or looking for a job (Table 4). People aged 18-29 years believed more often that psoriasis is contagious (p = 0.000). Similarly, females also tend to believe that psoriasis is contagious (p = 0.003). In parallel, regarding psoriasis and its relationship with hygiene, females and participants aged 18-29 years believed that psoriasis is caused by poor hygiene (p = 0.003 and p = 0.002, respectively). University graduates, compared to others, believed more often that psoriasis does not need lifelong treatment (p = 0.000). Participants with a monthly income of less than 10,000 SAR believed that psoriasis is a serious condition (p = 0.006). In the same manner, people aged 18-29 years believed that psoriasis is a serious condition (p = 0.000). Participants with a monthly income of less than 10,000 SAR and university graduates more often believed that psoriasis is not a genetic condition (p = 0.047 and p = 0.021, respectively) (Table 5).

**Table 4:** Participants’ negative prejudice toward psoriasis.

	Completely agree	Mostly agree	Completely disagree	Mostly disagree	Do not know
Psoriasis leads to a difficult love life	94 (11.7%)	158 (19.7%)	254 (31.6%)	170 (21.2%)	127 (15.8%)
Having psoriasis leads to problems in finding a job	100 (12.5%)	146 (18.2%)	177 (22%)	163 (20.3%)	217 (27%)
People with psoriasis tend to take more sick leave	107 (13.3%)	208 (25.9%)	131 (16.3%)	192 (23.9%)	165 (20.5%)

**Table 5:** Comparison between participants’ misconceptions and their demographics.

	Psoriasis is contagious	Psoriasis is due to poor hygiene		Psoriasis can be treated	Psoriasis needs life-long treatment	Psoriasis is a serious condition		Psoriasis is a genetic condition		Psoriasis can affect joints
Wrong answer	True	True		False	False	True		False		False
Total	76 (9.5%)	63 (7.8%)		111 (13.8%)	162 (20.2%)	141 (17.6%)		141 (17.6%)		157 (19.6%)
Age group (years)										
<18	6 (7.9%)	8 (12.7%)	9 (8.1%)		11 (6.8%)	11 (7.8%)	9 (6.4%)		5 (3.2%)	
18–29	33 (43.4%)	32 (50.8%)	52 (46.8%)		61 (37.7%)	72 (51.1%)	66 (46.8%)		50 (31.8%)	
30–39	13 (17.1%)	8 (12.7%)	17 (15.3%)		25 (15.4%)	15 (10.6%)	23 (16.3%)		25 (15.9%)	
40–49	11 (14.5%)	10 (15.9%)	9 (8.1%)		28 (17.3%)	18 (12.8%)	13 (9.2%)		35 (22.3%)	
50–60	10 (13.2%)	4 (6.3%)	21 (18.9%)		34 (21%)	23 (16.3%)	28 (19.9%)		35 (22.3%)	
>60	3 (3.9%)	1 (1.6%)	3 (2.7%)		3 (1.9%)	2 (1.4%)	2 (1.4%)		7 (4.5%)	
P-value	0.000	0.002	0.004		0.184	0.000	0.068		0.003	
Gender										
Male	17 (22.4%)	20 (31.7%)	32 (28.8%)		54 (33.3%)	39 (27.7%)	37 (26.2%)		42 (26.8%)	
Female	59 (77.6%)	43 (68.3%)	79 (71.2%)		108 (66.7%)	102 (72.3%)	104 (73.8%)		115 (73.2%)	
P-value	0.003	0.000	0.066		0.063	0.300	0.345		0.005	
Education level										
University	52 (68.4%)	42 (66.7%)	79 (71.2%)		103 (63.6%)	98 (69.5%)	98 (69.5%)		118 (75.2%)	
High school	21 (27.6%)	20 (31.7%)	31 (27.9%)		57 (35.2%)	40 (28.4%)	40 (28.4%)		36 (22.9%)	
Intermediate school	3 (3.9%)	1 (1.6%)	0 (0%)		1 (0.6%)	3 (2.1%)	2 (1.4%)		2 (1.3%)	
Elementary	0 (0%)	0 (0%)	1 (0.9%)		1 (0.6%)	0 (0%)	1 (0.7%)		1 (0.6%)	
P-value	0.462	0.552	0.016		0.000	0.753	0.021		0.420	
Monthly income										
Less than 10000 SAR	41 (53.9%)	40 (63.5%)	64 (57.7%)		80 (49.4%)	85 (60.3%)	82 (58.2%)		76 (48.4%)	
10000–20000 SAR	26 (34.2%)	15 (23.8%)	35 (31.5%)		60 (37%)	36 (25.5%)	47 (33.3%)		67 (42.7%)	
21000–30000 SAR	4 (5.3%)	4 (6.3%)	8 (7.2%)		14 (8.6%)	12 (8.5%)	6 (4.3%)		8 (5.1%)	
More than 30000 SAR	5 (6.6%)	4 (6.3%)	4 (3.6%)		8 (4.9%)	8 (5.7%)	6 (4.3%)		6 (3.8%)	
P-value	0.058	0.122	0.483		0.495	0.006	0.047		0.154	

## Discussion

This is the first study to determine the extent of the prevalence of negative prejudices, misconceptions, and discriminatory behavior in a Saudi Arabian community. A representative sample of the population of Jeddah city was used in our study to evaluate people’s perceptions of attitudes toward and behaviors related to psoriasis as well as the effects of age, gender, and education. Our study showed statistical evidence indicating that approximately 50% of Jeddah’s population held misconceptions about psoriasis and that 47% had negative prejudices toward those who have the condition. These findings should be taken into account from the viewpoint of society as they may explain the difficulties and, more specifically, the stigmatization that many psoriasis patients experience, which can occasionally result in psychological distress in those who do not express it. Therefore, to overcome the stigma arising from the general population avoiding physical contact with psoriasis patients and meeting other people’s eyes, patients must demonstrate adaptation or coping mechanisms. Such coping or adaptation strategies have been reported by Weiss et al. [15-17]. Our research emphasizes the widespread belief that psoriasis is contagious (9.5%) or caused by poor body hygiene (7.8%). This clear misunderstanding worsens the stigmatization that psoriasis patients already experience, making it harder for them. Misconceptions, negative prejudices, and discriminatory behavior toward psoriasis patients are strongly related. Ginsburg et al. estimated that 19% of psoriasis patients who exhibit such behavior have been requested to leave a location (a hair salon, a swimming pool, etc.) because of their condition [18]. Because approximately 34% of survey respondents still act in a way that is discriminatory toward people with psoriasis, the condition has significant implications for society. It is concerning that 18.7% and 14.1%, respectively, of respondents would hesitate and decline to shake hands with and sit close to people who have psoriasis. Notably, almost 30.7% of respondents believed that having psoriasis makes it difficult to find a job. Table 3 demonstrates that eating a meal prepared by a psoriasis patient was the least acceptable behavior. Only 34.5% would do it without hesitation and 13.3% completely rejected it, demonstrating the connection between meal preparation and a misleading belief that psoriasis is caused by poor hygiene. Our study showed that gender, age, and educational level are associated with an increased prevalence of misconceptions, as shown in Table 5, which demonstrates that people aged 18-29 years believed more often that psoriasis is contagious (p = 0.000). Similarly, females also believed that psoriasis is contagious (p = 0.003). In parallel, regarding psoriasis and its relationship with hygiene, females and participants aged 18-29 years believed that psoriasis is caused by poor hygiene (p = 0.003 and p = 0.002, respectively). University graduates, compared to others, believed more often that psoriasis does not require lifelong treatment (p = 0.000). While our results also showed that participants with a monthly income of less than 10,000 SAR believed that psoriasis is a serious condition (p = 0.006). Similarly, people aged 18-29 years believed that psoriasis is a serious condition (p = 0.000). On the other hand, participants with a monthly income of less than 10,000 SAR and university graduates more often believed that psoriasis is not a genetic condition (p = 0.047 and p = 0.021, respectively). While 56.5% of people in Jeddah said they would not shake hands with someone who had psoriasis, a previous survey done in France found the percentage to be 28.8%, which is significantly lower than that reported for Jeddah city alone and not Saudi Arabia [19]. In contrast, 16.6% and 6.8% of participants in France believed psoriasis to be contiguous and associated with poor hygiene, respectively. This contrasts with the 9.5% of participants in Jeddah who believed that psoriasis is contiguous and 7.8% who said that it is related to poor hygiene [19]. On the other hand, another study conducted in Malaysia among 164 participants showed that misconceptions were divided into poor hygiene (31.5%) as a cause of psoriasis. While 26.8% thought it was not a genetic condition, 16.5% thought it was not a serious condition. Regarding behavior, being friends with someone with psoriasis was acceptable by 67.7%, followed by sitting next to a psoriasis patient by 61.6%, and shaking hands by 42.9% [14].

The study’s use of an online poll, which might only reach a portion of people who are young and interested in technology, poses a potential limitation.

## Conclusions

Although our study was limited by the use of an online questionnaire in a limited location, it showed more favorable results on the general population’s perception and awareness of psoriasis regarding previously reported findings. However, the lack of information in the general population is undeniable, as our study demonstrated that the community needs more education on skin disorders, in general, and psoriasis, in particular. The worrisome lack of education about psoriasis in Jeddah causes patients with psoriasis to suffer more psychologically. Future studies should be undertaken in various ways and concentrate more on particular categories of people who physically engage with psoriasis patients, such as hairdressers, tailors, and massage therapists.
